# Preoperative fibrinogen-to-albumin ratio predicts the prognosis of patients with hepatocellular carcinoma subjected to hepatectomy

**DOI:** 10.1186/s12876-022-02328-4

**Published:** 2022-05-23

**Authors:** Rong-yun Mai, Tao Bai, Xiao-ling Luo, Guo-bin Wu

**Affiliations:** 1 Department of Hepatobilliary and Pancreatic Surgery, Guangxi Medical University Cancer Hospital, Nanning, 530021 China; 2 Department of Experimental Research, Guangxi Medical University Cancer Hospital, Nanning, 530021 China

**Keywords:** Hepatocellular carcinoma, Fibrinogen-to-albumin ratio, Prognosis, Systemic inflammation response

## Abstract

**Background:**

Systemic inflammatory response (SIR) plays a crucial role in every step of tumorigenesis and development. More recently, the fibrinogen-to-albumin ratio (FAR), an inflammation-based model, was suggested as a prognostic maker for various cancer patients. This research aimed to estimate the prognostic abilities of FAR, neutrophil–lymphocyte ratio (NLR), monocyte-lymphocyte ratio (MLR), platelet– lymphocyte ratio (PLR), and systemic immune–inflammation index (SII) in patients with hepatocellular carcinoma (HCC) subjected to curative hepatectomy.

**Methods:**

A total of 1,502 cases who underwent hepatectomy for HCC were included. The predictive performances of FAR, NLR, MLR, PLR and SII were assessed with regards to overall survival (OS) and disease-free survival (DFS). The area under the time-dependent receiver operating characteristic curve was used to compare prognostic performances.

**Results:**

Data revealed that FAR had higher predictive accuracy than other inflammation-based models and alpha-fetoprotein (AFP) in assessing OS and DFS. Indeed, the OS and DFS of patients with high FAR (> 8.9), differentiated by the optimal cut-off value of FAR, were remarkably reduced (*p* < 0.05 for OS and DFS). Multivariate Cox regression analyses identified that AFP, FAR, clinically significant portal hypertension, tumor size, Barcelona Clinical Liver Cancer staging system, major resection and blood loss were independent indicators for predicting OS and DFS. Furthermore, these patients could be classified according to their FAR into significantly different subgroups, regardless of AFP levels (*p* < 0.05 for DFS and OS). Similar results were obtained in other inflammation-based prognostic models.

**Conclusions:**

Compared with NLR, MLR, PLR, SII and AFP, FAR showed significant advantages in predicting survival of HCC patients subjected to liver resection.

**Supplementary Information:**

The online version contains supplementary material available at 10.1186/s12876-022-02328-4.

## Background

Hepatocellular carcinoma (HCC) is the sixth most common type of cancers and the fourth major leading cause of cancer-associated death worldwide [1]. Partial liver resection is the major strategy for the radical treatment of HCC [2, 3]. However, even after complete resection, the long-term patient survival rate remained unsatisfactory due to high recurrence rates [4, 5]. Therefore, an accurate and reliable model is needed to identify high-risk patients with poor prognoses and optimize their adjuvant therapy to achieve greater benefits.

Recently, systemic inflammation response (SIR) was shown to be an important distinguishing feature of malignant tumors since it is involved in every stage of tumorigenesis and development [4–6]. Many prognostic models based on the SIR have been established and are related the prognosis of patients with different cancers. These models usually consist of leukocytes and acute-phase proteins from the circulatory system. Currently, several leukocyte-based inflammation models, such as the neutrophil-to-lymphocyte ratio (NLR) [7–9], monocyte-to-lymphocyte ratio (MLR) [10, 11], platelet-to-lymphocyte ratio (PLR) [12, 13], and systemic immune– inflammation index (SII) [14, 15], have been proved to be meaningful indicators for estimating adverse results in various cancers. Nevertheless, these leukocytes are often affected by other factors and therefore they may not reflect the inflammatory state of patients [4]. For example, patients that receive transcatheter arterial chemoembolization, radiofrequency ablation, or immunotherapy, have significantly reduced levels of circulating platelets, lymphocytes, neutrophils and monocytes [6]. Therefore, these leukocyte-based models maybe have limitations in assessing the poor outcomes of patients undergoing hepatectomy for HCC.

Apart from inflammation, the nutritional status is also related to the outcomes of cancer patients [16, 17]. The modified Glasgow outcome scale (mGPS) [18, 19] and C-reactive protein-to-albumin ratio (CAR) [20], are based on the two important acute phase proteins in SIR, namely C-reactive protein (CRP) and albumin [4], and are related to the prognosis of cancer patients. Nevertheless, in many treatment centers, CRP is not included as a routine measurement of patient care, which limits its clinical application [21]. In parallel, fibrinogen, a glycoprotein synthesized by liver cells, is the most abundant coagulation parameter in humans and is routinely measured [22]. Interestingly, plasma fibrinogen is also an acute phase protein in SIR, similar to CPR, which increases rapidly during inflammation [22]. After being converted to fibrin, it is actively involved in the coagulation process. Many researchers have confirmed that increased levels of plasma fibrinogen may predict adverse events for various cancers [21–25]. In addition, fibrinogen has been shown to have the same predictive power as CRP in assessing adverse outcomes in cancer patients [22]. Therefore, the fibrinogen-to-albumin ratio (FAR) [26–32], which is composed of plasma fibrinogen and serum albumin, combines the patient's inflammation, coagulation and nutritional situation, and may be a valuable model to assess the poor outcomes of HCC patients.

Therefore, the purpose of this research was to estimate the capacity of FAR in the prognosis prediction of patients with HCC subjected to hepatectomy. Moreover, FAR was compared with other models, including NLR, MLR, PLR and SII.

## Patients and methods

### Patients

This study included all HCC patients who were subjected to hepatic resection with curative intent at Guangxi Medical University Cancer Hospital between December 2013 and December 2018. Patients who received other therapies for HCC before hepatectomy were excluded. The study was conducted in compliance with the Helsinki Declaration and approved by the institutional Ethics Committee of Guangxi Medical University Cancer Hospital, and all patients provided written informed consent.

### Diagnosis and definitions

Cirrhosis and HCC were diagnosed on the based of postoperative histological evidence. HCC staging was performed based on the Barcelona Clinical Liver Cancer (BCLC) staging System [33]. Clinically significant portal hypertension (CSPH) was defined as splenomegaly or gastroesophageal varices with thrombocytopenia [34]. Major hepatectomy was defined as the resection of 3 or more segments of the liver [35].

### Calculation of systemic inflammation-based prognostic models

The prognostic models were calculated as: FAR = fibrinogen (mg/dL)/albumin (mg/dL) × 100; MLR = monocyte count/lymphocyte counts; NLR = neutrophil counts/lymphocyte counts; PLR = platelet counts/lymphocyte counts; SII = platelet counts × neutrophil counts/lymphocyte counts. Measurements were performed one week before hepatectomy. All indicators of the above formula were collected and analyzed within 1 week prior to hepatectomy.

### Surgical procedure and follow-up

All patients included in this project were performed open hepatectomy when preoperative imaging showed that tumors could be removed with a good liver function reserve. Further details and indications of surgery were described in previous research [36].

After discharge, follow-up was performed 1 month after liver resection and every 3 months until withdrawal or death. At each follow-up, liver function assay, serum alpha-fetoprotein (AFP) levels and abdominal CT or MRI scans were performed. Patients with tumor recurrence were subjected to radiofrequency, chemotherapy, re-operation or targeted therapy as appropriate. Overall survival (OS) refers to the time between the date of hepatectomy and the last follow-up or death, while disease-free survival (DFS) refers to the time between the date of hepatectomy and the first recrudescence or no relapse at the last follow-up [37].

### Statistical analysis

Continuous data are shown as medians (IQR 25–75) and were compared by Mann–Whitney U tests. Categorical factors are expressed as n (%) and were compared using χ^2^ tests.

The time-dependent receiver operating characteristic (t-ROC) curves was applied to confirm the discriminatory performance of inflammation-based prognostic models in assessing OS and DFS [38]. The optimal cut-off value was confirmed by X-tile analysis of the 5-year OS [39]. OS and DFS were evaluated by the Kaplan–Meier (KM) method, and discrepancies among groups were compared by the log-rank test. A Cox regression model was used for the multivariable analysis to confirm independent prognostic variables of OS and DFS. Indicators with p < 0.05 in univariate analyses were added to the multivariate Cox analysis.

RStudio (v1.4.1106), X-Tile (v3.6.1) and SPSS (v25.0) software were used for corresponding statistical analyses, and *p* < 0.05 was considered to be statistically significant.

## Results

### Patient characteristics

The clinicopathologic features of 1,502 HCC cases were shown in Table 1. The patient population had a medians age of 50 years, and 86.0% of them were male. The majority of patients (86.0%) suffered from hepatitis B virus infection, 43.3% had liver cirrhosis and 5.4% had CSPH. Most of the patients (93.3%) had good liver reserve function, and only 6.7% of the patients were Child–Pugh B grade. The medians of the prognostic indexes were 6.9 (IQR 5.5–8.9) for FAR, 2.1 (IQR 1.5–3.0) for NLR, 0.3 (IQR 0.2–0.4) for MLR, 115.7 (IQR 85.4–160.6) for PLR, and 422 (IQR 262–682) for SII. Based on the BCLC grading, 3.5% of patients were grade 0, 52.3% were grade A, 17.1% were grade B, and 27.1% were grade C. Surgical hepatectomies included 754 major and 748 minor hepatectomies.

**Table 1:** Baseline characteristics of 1502 HCC patients and patients with high and low FAR risk groups

Variables	Total (n = 1502)	Low FAR (n = 1126)	High FAR (n = 376)	*P* value
Age(years)	50 (43, 59)	50 (42, 59)	52 (45, 60)	0.017
*Sex*				0.242
Male	1291 (86.0)	961 (85.3)	330 (87.8)	
Female	211 (14.0)	165 (14.7)	46 (12.2)	
Positive HBsAg	1297 (86.4)	979 (86.9)	318(84.6)	0.246
Positive anti-HCV	22 (1.5)	18 (1.6)	4 (1.1)	0.455
Platelet count (10^9^/L)	207 (160, 266)	192 (149, 243)	263 (211, 336)	< 0.001
Neutrophil count (10^9^/L)	3.6 (2.8, 4.8)	3.3 (2.6, 4.4)	4.5 (3.6, 5.9)	< 0.001
Lymphocyte count (10^9^/L)	1.7 (1.4, 2.2)	1.8 (1.4, 2.2)	1.7 (1.3, 2.1)	< 0.001
Monocyte count (10^9^/L)	0.5 (0.4, 0.6)	0.4 (0.3, 0.6)	0.6 (0.4, 0.8)	< 0.001
Total bilirubin (μmol/L)	13.1 (9.7, 17.9)	13.2 (9.9, 17.8)	12.8 (9.4, 18.2)	0.615
Albumin (g/L)	39.4 (36.2, 42.4)	40.3 (37.4, 43.3)	35.9 (33.5, 39.2)	< 0.001
ALT (U/L)	35 (24, 50)	35 (24, 50)	34 (22, 54)	0.813
AST (U/L)	39 (29, 57)	37 (29, 54)	46 (32, 69)	< 0.001
Prothrombin time (s)	12.8 (12.0, 13.6)	12.7 (12.0, 13.6)	12.9 (12.2, 13.9)	0.106
Fibrinogen (g/L)	2.7 (2.2, 3.4)	2.5 (2.1, 2.9)	4.0 (3.5, 4.7)	< 0.001
*Child–Pugh grade*				< 0.001
A	1401 (93.3)	1076 (95.6)	325 (86.4)	
B	101 (6.7)	50 (4.4)	51 (13.6)	
*AFP ( ng/mL)*				0.449
≥ 400	638 (42.5)	472 (41.9)	166 (44.1)	
< 400	864 (57.5)	654 (58.1)	210 (55.9)	
FAR	6.9 (5.5, 8.9)	6.2 (5.2, 7.3)	10.9 (9.6, 13.1)	< 0.001
NLR	2.1 (1.5, 3.0)	1.9 (1.4, 2.5)	2.9 (2.0, 4.1)	< 0.001
PLR	115.7 (85.4, 160.6)	104.6 (79.5, 141.7)	157.0 (116.3, 210.5)	< 0.001
MLR	0.3 (0.2, 0.4)	0.2 (0.2, 0.3)	0.3 (0.3, 0.5)	< 0.001
SII	422 (262, 682)	354 (229, 543)	744 (485, 1156)	< 0.001
CSPH	136 (9.1)	111 (9.9)	25 (6.6)	0.060
Ascites	169 (11.3)	106 (9.4)	63 (16.8)	< 0.001
Cirrhosis	680 (45.3)	527 (46.8)	153 (40.7)	0.039
*Tumour size*				< 0.001
> 5	883 (58.8)	570 (50.6)	313 (83.2)	
≤ 5	619 (41.2)	556 (49.4)	63 (16.8)	
*Tumour number*				0.035
Multiple	288 (19.2)	202 (17.9)	86 (29.9)	
Single	1214 (80.8)	924 (82.1)	290 (77.1)	
MVI	393 (26.2)	241 (21.4)	152 (38.7)	< 0.001
*BCLC stage*				< 0.001
0	53 (3.5)	51 (4.5)	2 (0.5)	
A	785 (52.3)	633 (56.2)	152 (40.4)	
B	257 (17.1)	192 (17.1)	65 (17.3)	
C	407 (27.1)	250(22.2)	157 (41.8)	
*Blood loss (mL)*				< 0.001
≥ 400	541 (36.0)	366 (32.5)	175 (46.5)	
< 400	961 (64.0)	760 (67.5)	201 (53.5)	
Blood transfusion	198 (13.2)	123 (10.9)	75 (19.9)	< 0.001
*Extent of resection*				< 0.001
Major hepatectomy	754 (50.2)	513 (45.6)	241 (64.1)	
Minor hepatectomy	748 (49.8)	613 (54.4)	135 (35.9)	

Continuous data are show as median (25th–75th interquartile range) and categorical data are expressed as n (%)

*HCC*, hepatocellular carcinoma; *HBsAg*, hepatitis B surface antigen; *HCV*, hepatitis C virus; *ALT*, Alanine aminotransferase; *AFP*, alpha-fetoprotein; *FAR*, fibrinogen-albumin ratio; *NLR*, neutrophil–lymphocyte ratio; *MLR*, monocyte-lymphocyte ratio; *PLR*, platelet–lymphocyte ratio; *SII*, systemic immune–inflammation index; *CSPH*, clinically significant portal hypertension; *MVI*, macrovascular invasion; *BCLC*, Barcelona Clinical Liver Cancer

### Discriminatory performance of inflammation-based prognostic models

**Fig. 1:**
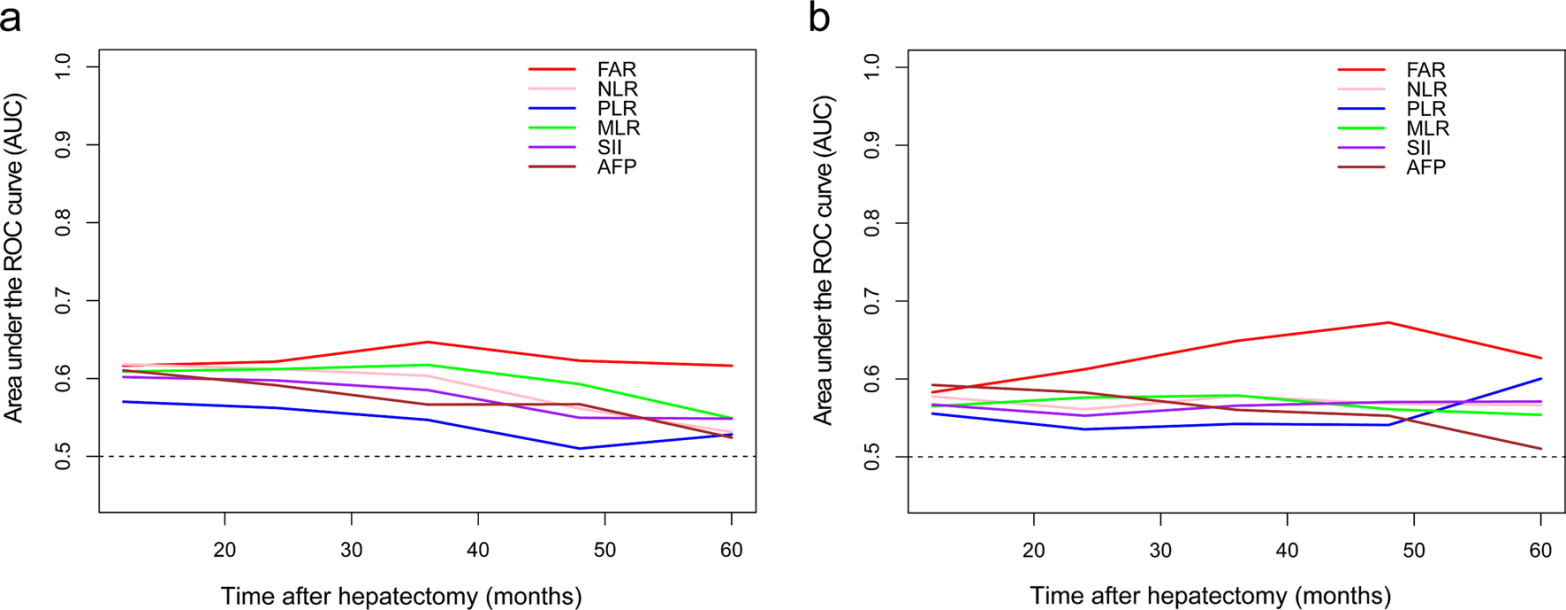
Time-dependent receiver operating characteristic curve analysis to compare the efficacy of FAR and other models in predicting **a** OS and **b** DFS. FAR, fibrinogen-albumin ratio; NLR, neutrophil–lymphocyte ratio; MLR, monocyte-lymphocyte ratio; PLR, platelet–lymphocyte ratio; SII, systemic immune–inflammation index; AFP, alpha-fetoprotein; OS overall survival; DFS disease-free survival

Upon the analysis of t-ROC curves, FAR was found to have a higher capacity to assess OS when compared with the ability of other inflammation-based prognostic models, including NLR, MLR, PLR and SII (Fig. 1a). Similarly, the area under t-ROC curve (AUC) of FAR in evaluating DFS were also greater than that of other models at every time point after liver resection (Fig. 1b). Furthermore, the prediction performance of FAR for OS and DFS was also higher than that of AFP. FAR values predicted that the AUC of 1-, 3- and 5- years OS were respectively 0.622, 0.632 and 0.640, while those of DFS were respectively 0.588, 0.641 and 0.666 (Additional File 1: Table S1).

### The optimal cut-off value of inflammation-based prognostic models

**Fig. 2:**
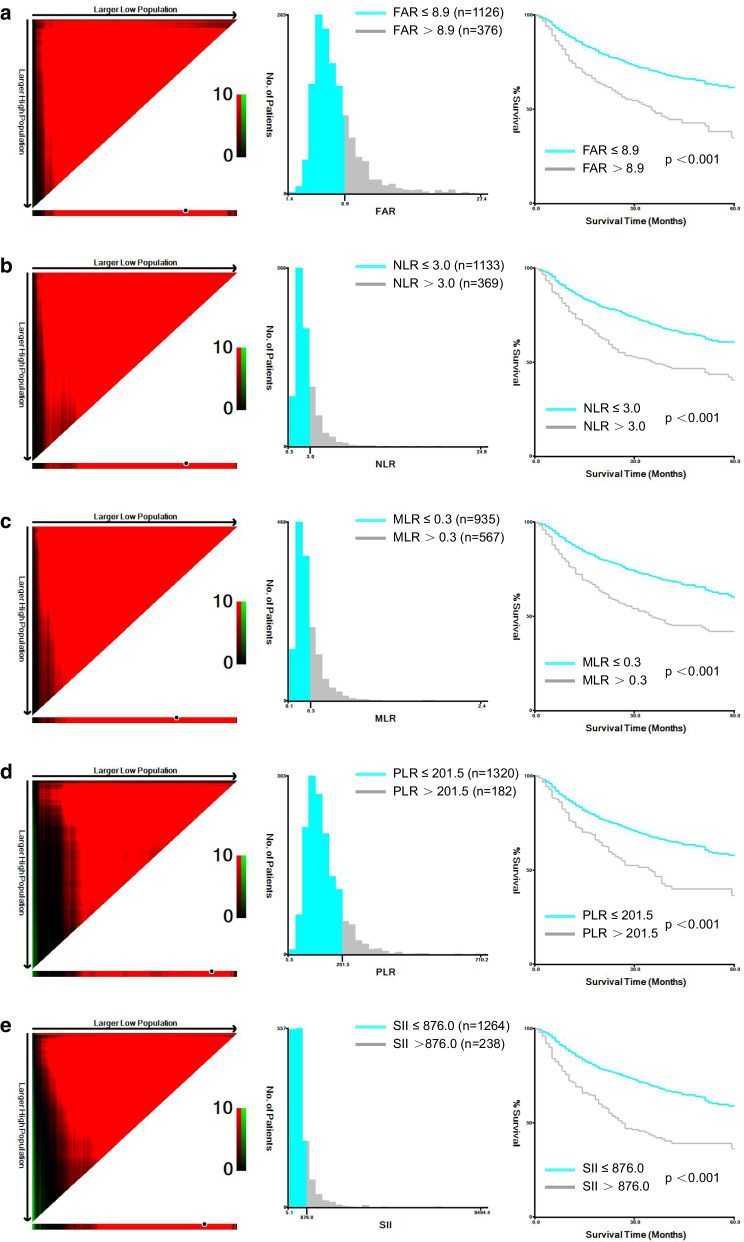
X-tile analyses to determine the optimal cut-off values of **a** FAR, **b** NLR, **c** MLR, **d** PLR and **e** SII. The optimal cut-off values of FAR, NLR, MLR, PLR and SII were 8.9, 3.0, 0.3, 201.5, and 876.0, respectively. FAR, fibrinogen-albumin ratio; NLR, neutrophil–lymphocyte ratio; MLR, monocyte-lymphocyte ratio; PLR, platelet–lymphocyte ratio; SII, systemic immune–inflammation index; AFP, alpha-fetoprotein

We used the X-tile software to identify the optimal cut-off values of these models. As shown in Fig. 2, the values for FAR, NLR, MLR, PLR, and SII were 8.9, 3.0, 0.3, 201.5, and 876.0, respectively. Moreover, all patients were classified into low (≤ 8.9, n = 1126) or high (> 8.9, n = 376) FAR groups for further analysis according to this value of FAR.

### Correlation between FAR and clinicopathological variables

As presented in Table 1, patients in the high FAR cohort were older, had higher serum levels (platelet, neutrophil, lymphocyte counts, etc.), lower serum albumin levels, worse liver background (higher frequency of Child–Pugh grade B, cirrhosis and CSPH), worse tumor condition (larger tumor size, multiple tumors, with macrovascular invasion (MVI) and advanced BCLC staging), and worse surgical environment (greater blood loss and transfusions, more major resections). In addition, the values of NLR, MLR, PLR and SII were also greatly up-regulated in the high FAR set (p < 0.05 for all).

### Comparison of OS and DFS based on FAR

**Fig. 3:**
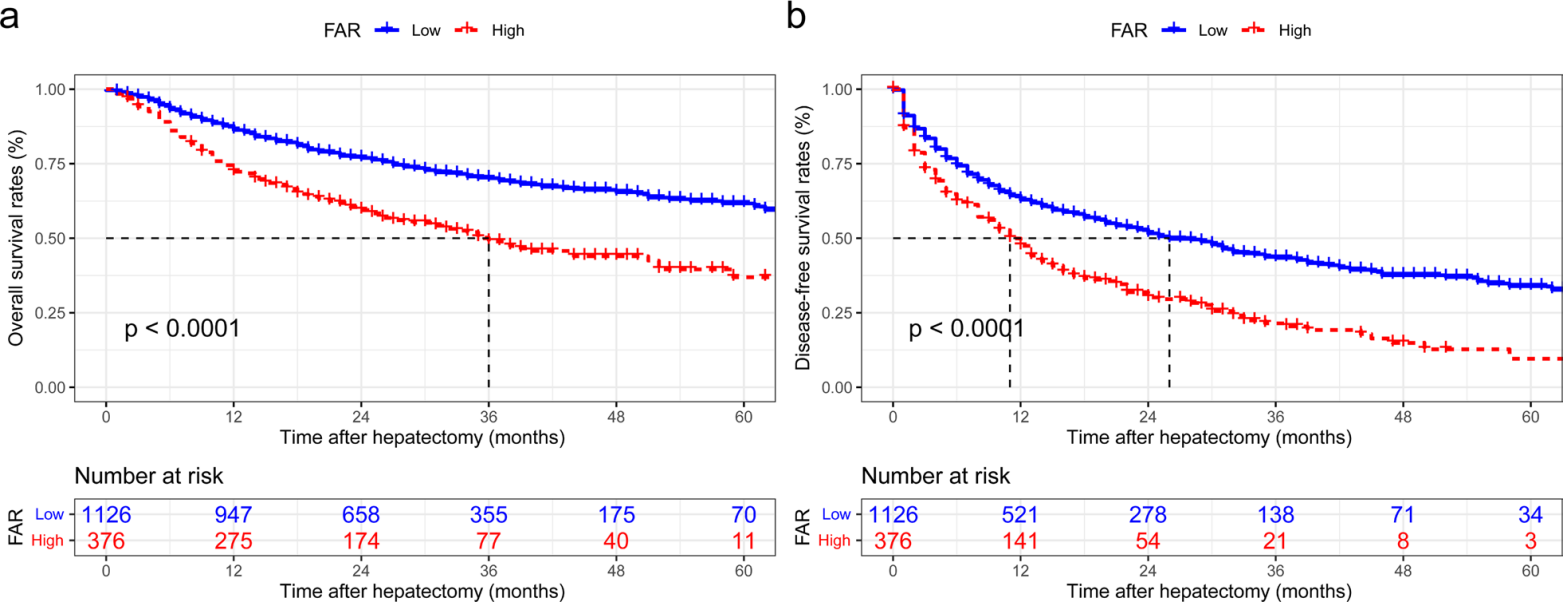
Kaplan–Meier curves illustrating **a** OS (p < 0.001) and **b** DFS (p < 0.001) based on different risk groups of FAR in the entire cohort. OS overall survival; DFS disease-free survival; FAR, fibrinogen- albumin ratio

During a median follow-up of 26 months (15–38), 321 cases (28.5%) died in the low FAR group, while 179 patients (47.6%) died in the high FAR group (p < 0.05). The 1-, 3-, and 5-year OS of the high FAR set were respectively 72.3%, 48.9%, and 36.9%, which was remarkably worse than that of the low FAR group (86.3%, 70.2%, and 61.7%, respectively; *p* < 0.05 for all; Fig. 3a). Furthermore, tumor recurrence occurred in 719 patients (47.8%) during follow-up, including 787 patients (43.3%) in the low FAR group and 232 patients (61.7%) in the high FAR group (*p* < 0.05). Among these patients, 139 patients (23.4%) had an extrahepatic relapse, while most of the remaining patients (85.6%) had an intrahepatic recurrence. The 1-, 3-, and 5-year DFS of patients in the high FAR set were 47.4%, 20.4%, and 9.6%, respectively, which were also remarkably worse than those of the low FAR group (62.9%, 43.4%, and 33.9%, respectively; *p* < 0.05 for all; Fig. 3b).

### Independent predictors of OS

In univariable Cox analyses, FAR, NLR, MLR, PLR and SII were significantly related to OS, as well as age, male sex, ascites, AFP, cirrhosis, tumor size, macrovascular invasion, BCLC stage, blood loss, blood transfusion and major hepatectomy. The multivariable Cox analysis revealed that FAR (HR 1.230, 95% CI 1.001–1.510; *p* = 0.048) was an independent risk index of OS. Other independent prognostic factors included AFP, PLR, SII, CSPH, tumor size and blood loss (Table 2).

**Table 2:** Univariable and multivariable analyses to identify independent prognostic indicators of overall survival in pateints with HCC

Variables	Overall survival			
	Univariable		Multivariable	
	HR (95% CI)	*p* value	HR (95% CI)	*p* value
Age (years)	0.988 (0.980, 0.996)	0.003	0.992 (0.984,1.000)	0.050
Male sex	1.442 (1.085, 1.917)	0.012	1.293 (0.967, 1.729)	0.083
Positive HBsAg	1.320 (0.995, 1.751)	0.054		
Positive anti-HCV	0.634 (0.263, 1.531)	0.311		
Child–Pugh grade B	1.340 (0.962, 1.886)	0.084		
AFP ≥ 400 ng/mL	1.767 (1.482, 2.107)	< 0.001	1.346 (1.120, 1.618)	0.002
FAR	1.961 (1.632, 2.355)	< 0.001	1.230 (1.001, 1.510)	0.048
NLR	1.936 (1.611, 2.326)	< 0.001	1.045 (0.801, 1.362)	0.748
PLR	1.839 (1.466, 2.307)	< 0.001	0.739 (0.548, 0.998)	0.049
MLR	1.945 (1.632, 2.319)	< 0.001	1.166 (0.940, 1.446)	0.163
SII	2.210 (1.809, 2.700)	< 0.001	1.455 (1.053, 2.010)	0.023
CSPH	1.390 (1.050, 1.840)	0.021	1.369 (1.022, 1.833)	0.035
Ascites	1.471 (1.149, 1.883)	0.002	1.186(0.914, 1.539)	0.198
Cirrhosis	1.085 (0.910, 1.294)	0.364		
Tumor size ≥ 5 cm	2.708 (2.200, 3.334)	< 0.001	1.500 (1.180, 1.907)	0.001
Multiple number	1.093 (0.871, 1.371)	0.441		
MVI	3.484 (2.920, 4.156)	< 0.001	1.522 (0.711, 3.256)	0.279
BCLC stage C	8.773 (3.619, 21.267)	< 0.001	2.693 (0.835, 8.686)	0.097
Blood loss ≥ 400 ml	2.028(1.701, 2.417)	< 0.001	1.232 (1.011, 1.501)	0.039
Blood transfusion	1.554 (1.225, 1.971)	< 0.001	1.083 (0.843, 1.392)	0.534
Major hepatectomy	1.792 (1.498, 2.143)	< 0.001	1.075 (0.881, 1.311)	0.477

*HCC*, hepatocellular carcinoma; *HBsAg*, hepatitis B surface antigen; *HCV*, hepatitis C virus; *AFP*, alpha-fetoprotein; *FAR*, fibrinogen-albumin ratio; *NLR*, neutrophil–lymphocyte ratio; *MLR*, monocyte-lymphocyte ratio; *PLR*, platelet–lymphocyte ratio; *SII*, systemic immune–inflammation index; *CSPH*, clinically significant portal hypertension; *MVI*, macrovascular invasion; *BCLC*, Barcelona Clinical Liver Cancer

### Independent predictors of DFS

Univariable Cox analyses revealed that these inflammation-based prognostic models (FAR, NLR, MLR, PLR, and SII) were significantly related to DFS, as well as age, male sex, HBsAg, AFP, CSPH, ascites, tumor size, BCLC stage, blood loss and transfusion, and major hepatectomy. In the multivariable Cox analysis, FAR (HR 1.211, 95% CI 1.014–1.445; *p* = 0.035) was shown as an independent predictive parameter of DFS, followed closely by HBsAg, AFP, CSPH, tumor size, BCLC stage and blood loss (Table 3).

**Table 3:** Univariable and multivariable analyses to identify independent prognostic indicators of disease-free survival in patients with HCC

Variables	Disease-free survival			
	Univariable		Multivariable	
	HR (95% CI)	*p* value	HR (95% CI)	*p* value
Age (years)	0.988 (0.982, 0.994)	< 0.001	0.994 (0.987, 1.001)	0.078
Male sex	1.292 (1.033, 1.616)	0.025	1.170 (0.927, 1.478)	0.187
Positive HBsAg	1.603 (1.259, 2.041)	< 0.001	1.509 (1.167, 1.951)	0.002
Positive anti-HCV	0.775 (0.415, 1.447)	0.424		
Child–Pugh grade B	1.167 (0.870, 1.565)	0.302		
AFP ≥ 400 ng/mL	1.560 (1.351, 1.801)	< 0.001	1.277 (1.095, 1.489)	0.002
FAR	1.534 (1.311, 1.796)	< 0.001	1.211 (1.014, 1.445)	0.035
NLR	1.542 (1.318, 1.803)	< 0.001	1.086 (0.866, 1.362)	0.474
PLR	1.646 (1.350, 2.008)	< 0.001	0.970 (0.744, 1.265)	0.821
MLR	1.453 (1.257, 1.679)	< 0.001	1.055 (0.878, 1.267)	0.568
SII	1.676 (1.402, 2.005)	< 0.001	1.143 (0.857, 1.524)	0.364
CSPH	1.306 (1.033, 1.652)	0.026	1.319 (1.030, 1.689)	0.028
Ascites	1.303 (1.054, 1.610)	0.014	1.129 (0.903, 1.411)	0.287
Cirrhosis	1.150 (0.996, 1.328)	0.056		
Tumor size ≥ 5 cm	2.024 (1.732, 2.364)	< 0.001	1.348 (1.121, 1.620)	0.001
Multiple number	1.057 (0.876, 1.275)	0.565		
MVI	2.222 (1.910, 2.584)	< 0.001	0.829 (0.424, 1.622)	0.584
BCLC stage C	4.951 (2.835, 8.646)	< 0.001	3.109 (1.303, 7.416)	0.011
Blood loss ≥ 400 ml	1.860 (1.609, 2.149)	< 0.001	1.338 (1.136, 1.575)	< 0.001
Blood transfusion	1.312 (1.056, 1.629)	0.014	0.951 (0.755, 1.200)	0.674
Major hepatectomy	1.663 (1.438, 1.924)	< 0.001	1.144 (0.972, 1.346)	0.106

*HCC*, hepatocellular carcinoma; *HBsAg*, hepatitis B surface antigen; *HCV*, hepatitis C virus; *AFP*, alpha-fetoprotein; *FAR*, fibrinogen-albumin ratio; *NLR*, neutrophil–lymphocyte ratio; *MLR*, monocyte-lymphocyte ratio; *PLR*, platelet–lymphocyte ratio; *SII*, systemic immune–inflammation index; *CSPH*, clinically significant portal hypertension; *MVI*, macrovascular invasion; *BCLC*, Barcelona Clinical Liver Cancer

### Subgroup analyses

**Fig. 4:**
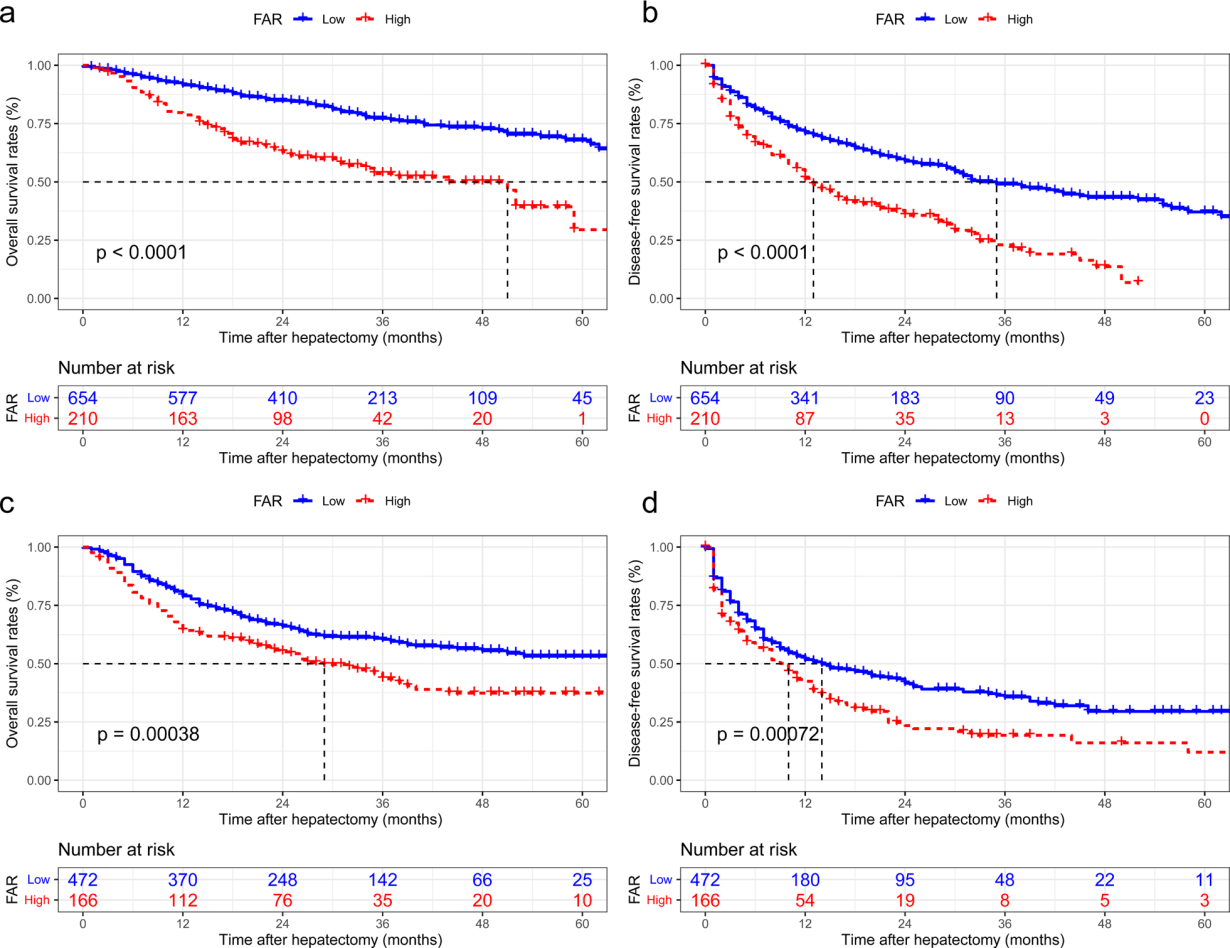
The OS and DFS of patients with different risk groups of FAR were subgroup analyzed according to AFP level. **a** OS in patients with low AFP (p < 0.001); **b** DFS in patients with low AFP (p < 0.001); **(c)** OS in patients with high AFP (p < 0.001); and **d** DFS in patients with high AFP (p < 0.001). OS overall survival; DFS disease-free survival; FAR, fibrinogen- albumin ratio; AFP, alpha-fetoprotein

Using the cut-off value of 8.9, patients with low AFP were divided into two distinct subgroups with remarkably disparate OS and DFS (*p* < 0.001 for all, Fig. 4a, b). Similarly, OS and DFS in the high FAR subgroup were expressively worse than those in the low FAR subgroup for patients with high AFP (*p* < 0.001 for all, Fig. 4c, d). Furthermore, low and high FAR were also divided according to NLR and MLR into two subgroups with meaningfully different OS and DFS (Additional File 1: Figs. S1 and S 2). As for PLR and SII, there were significant differences in each subgroup, except for DFS in the high PLR group, as well as OS and DFS in the high SII group (Additional File 1: Figs. S3 and S4).

## Discussion

In this study, we compared differences in the prognostic ability of FAR, NLR, MLR, PLR and SII in HCC patients subjected to hepatectomy. The discriminatory performance of FAR was found to be greater than that of other prognostic models. In addition, preoperative FAR was an independent prediction parameter of OS and DFS. When compared with AFP, FAR showed a higher predictive value and consistent prognostic ability for patients with disparate clinicopathological features. Therefore, FAR is a meaningful inflammation-based predictor in patients with HCC subjected to liver resection.

Currently, many researchers have suggested that SIR is closely connected with tumorigenesis and tumor development [4–6]. Interestingly, fibrinogen is not only an acute phase reactive protein that reflects SIR, but also a vital index involved in maintaining hemostasis, and the hemostasis system also plays a key effect in the occurrence and development of malignancies [40]. According to its ability to directly bind to members of the transforming growth factor-β, vascular endothelial growth factor, fibroblast growth factor and platelet-derived growth factor, fibrinogen plays a vital role in angiogenesis, epithelial-tomesenchymal transition, proliferation, and metastasis of cancer cells [40, 41]. Moreover, studies have shown that increased plasma fibrinogen is positively correlated with low survival rate of cancer patients [42], and adjuvant therapy that down-regulates plasma fibrinogen level may effectively prolong long-term survival of cancer patients [43]. Albumin is a commonly applied indicator to evaluate a patient's nutritional and immune status, and it can stabilize DNA replication and enhance the immune response, thereby inhibiting tumor progression [44]. In addition, reduced serum albumin is often closely related to poor outcomes in cancer patients [45]. FAR plays an mechanistically vital role in SIR, coagulation and nutrition, and is closely related to the increased metastatic potential caused by tumor cell survival, adhesiveness and intraversion [46]. These may be the critical reasons and mechanisms why FAR can be a powerful prognostic factor for cancer patients.

In this study, t-ROC analyses showed that FAR was greatly better than other inflammation prognostic models, including NLR, MLR, PLR, and SII, in estimating postoperative outcomes of patients with HCC. The optimum cut-off value of FAR was determined to be 8.9 by the X-tile analysis. High FAR (> 8.9) was more possibly to happen in patients with worse cancer condition (e.g. larger tumor size, multiple tumors, accompanied by MVI and advanced BCLC staging), which suggests that FAR may reflect HCC progression and metastasis. Through a survival analysis, we found that OS and DFS were remarkably worse in patients with high FAR, as compared with patients with low FAR. In addition, univariable and multivariable analyses determined that high FAR was an independent predictive indicator for OS and DFS. Thus, these results suggest that FAR can be used as a satisfactory prognostic tool for HCC patients subjected to hepatic resection.

Other inflammation-based prognostic models were evaluated and compared to FAR, but did not show independent prognostic value. Previous studies have shown that NLR can independently predict poor outcomes of HCC patients who underwent hepatectomy. However, in our research, NLR was not found to be an independent predictor, which is similar to results from the United Kingdom [47]. In parallel, Kaida et al. [12] found that preoperative PLR could predict the recurrence of HCC in patients who exceeded the Milan criteria after liver resection. Moreover, Yang et al. [48] showed that preoperative PLR was a negative survival indicator after liver resection for HBV-related HCC. We found that high PLR was also related to poor OS and DFS, even though DFS was not significant in the multivariate analysis, and there was no advantage over other models in predicting prognosis. Similar results were found for MLR and SII. The poor prognostic ability of these models in the multivariate analysis may be due to their association with FAR. In addition, other prognostic models showed weaker prognostic effects when compared with FAR. Therefore, we conclude that FAR was a better prognostic predictor in patients with HCC subjected to liver resection, when compared with existing inflammation-based prognosis models.

Recently, AFP is the most commonly applied index to evaluate the poor outcomes of HCC patients. In this research, AFP was confirmed as an independent predictive marker of OS and DFS in HCC patients subjected to liver resection. Surprisingly, t-ROC analyses showed that FAR had higher discriminatory performance than AFP in both OS and DFS. A subgroup analysis in which cases were divided according to both their FAR and AFP was performed. This analysis suggested that, compared with AFP, FAR was a better model for estimating the prognosis of HCC patients undergoing hepatectomy. Similar results have been obtained in other models. Considering that patients with high FAR presented poor prognosis, perioperative adjuvant therapies may assist to decrease the recurrence risk in these patients, prolong survival and improve quality of life. Moreover, closer follow-up may be considered so that recurrence can be detected earlier in these patients and then treated as soon as possible.

However, this study also have some limitations. Firstly, most patients suffered from HBV-related HCC, thus, the prognostic feasibility of FAR in patients with HCC due to other causes needs more evidence. Secondly, because our center does not detect CRP in routine blood tests before surgical operations, this study did not analyze inflammation markers related to CRP. Furthermore, this was a retrospective and single-center research, so further researches are required to ascertain our findings.

## Conclusion

Preoperative FAR can more accurately predict OS and DFS than previously established inflammation-based prognostic models and AFP in patients undergoing hepatectomy for HCC. Its availability, low cost and convenience makes FAR a promising inflammation-based tool to assess the prognosis of HCC patients and may support future treatment decisions.

## Supplementary Information

**Additional file 1:** Assessed AUCs of inflammation-based prognostic models and AFP to predict OS and DFS, and subgroups analyses of OS and DFS in different FAR risk groups based on these models.

## Data Availability

The datasets used and/or analysed during the current study are available from the corresponding author on reasonable request.

## Abbreviations

HCC: Hepatocellular carcinoma
SIR: Systemic inflammation response
OS: Overall survival
DFS: Disease-free survival
FAR: Fibrinogen-to-albumin ratio
NLR: Neutrophil–lymphocyte ratio
MLR: Monocyte-lymphocyte ratio
PLR: Platelet–lymphocyte ratio
SII: Systemic immune–inflammation index
mGPS: Modified Glasgow outcome scale
CAR: C-reactive protein-to-albumin ratio
CRP: C-reactive protein
AFP: α-Fetoprotein
MVI: Macrovascular invasion
CSPH: Clinically significant portal hypertension
BCLC: Barcelona Clinical Liver Cancer
t-ROC: Time-dependent receiver operating characteristic
