# Supplementary material for: Preoperative fibrinogen-to-albumin ratio predicts the prognosis of patients with hepatocellular carcinoma subjected to hepatectomy
Source: BMC Gastroenterol. 2022 May 23;22:261. doi: 10.1186/s12876-022-02328-4 (PMC9128092; doi:10.1186/s12876-022-02328-4)
Supplement: Supplementary file 1 — Additional file 1: Assessed AUCs of inflammation-based prognostic models and AFP to predict OS and DFS, and subgroups analyses of OS and DFS in different FAR risk groups based on these models. [file 12876_2022_2328_MOESM1_ESM.docx]

**Supplemental contents to:**

**Preoperative fibrinogen-to-albumin ratio predicts the prognosis of patients with hepatocellular carcinoma subjected to hepatectomy**

*Rong-yun Mai M.D., Tao Bai M.D., Xiao-ling Luo PH.D., Guo-bin Wu M.D.*

**Table of Contents**

**Supplementary Table 1**. Assessed AUCs of inflammation-based prognostic models and AFP in predicting OS and DFS at various time points in patients with HCC 2

**Supplementary Fig 1**. The OS and DFS of patients with high and low FAR were subgroup analyzed according to NLR level 3

**Supplementary Fig 2**. The OS and DFS of patients with high and low FAR were subgroup analyzed according to MLR level. 4

**Supplementary Fig 3.** The OS and DFS of patients with high and low FAR were subgroup analyzed according to MLR level. 5

**Supplementary Fig 4.** The OS and DFS of patients with high and low FAR were subgroup analyzed according to SII level. 6

Supplementary table 1. Assessed AUCs of inflammation-based prognostic models and AFP in predicting OS and DFS at various time points in patients with HCC

| Time after hepatectomy | AUC (t) | | | | | |
| --- | --- | --- | --- | --- | --- | --- |
|  | NLR | PLR | MLR | SII | AFP | FAR |
| **OS** |  |  |  |  |  |  |
| 1-year | 0.618 | 0.577 | 0.617 | 0.607 | 0.613 | **0.622** |
| 3-years | 0.606 | 0.563 | 0.618 | 0.593 | 0.582 | **0.632** |
| 5-years | 0.596 | 0.566 | 0.603 | 0.592 | 0.554 | **0.640** |
| **DFS** |  |  |  |  |  |  |
| 1-year | 0.58 | 0.561 | 0.568 | 0.573 | 0.586 | **0.588** |
| 3-years | 0.580 | 0.570 | 0.577 | 0.570 | 0.560 | **0.641** |
| 5-years | 0.569 | 0.580 | 0.560 | 0.560 | 0.539 | **0.666** |

Abbreviations: HCC, hepatocellular carcinoma; AFP, alpha-fetoprotein; FAR, fibrinogen-albumin ratio; NLR, neutrophil–lymphocyte ratio; MLR, monocyte-lymphocyte ratio; PLR, platelet–lymphocyte ratio; SII, systemic immune–inflammation index.

**
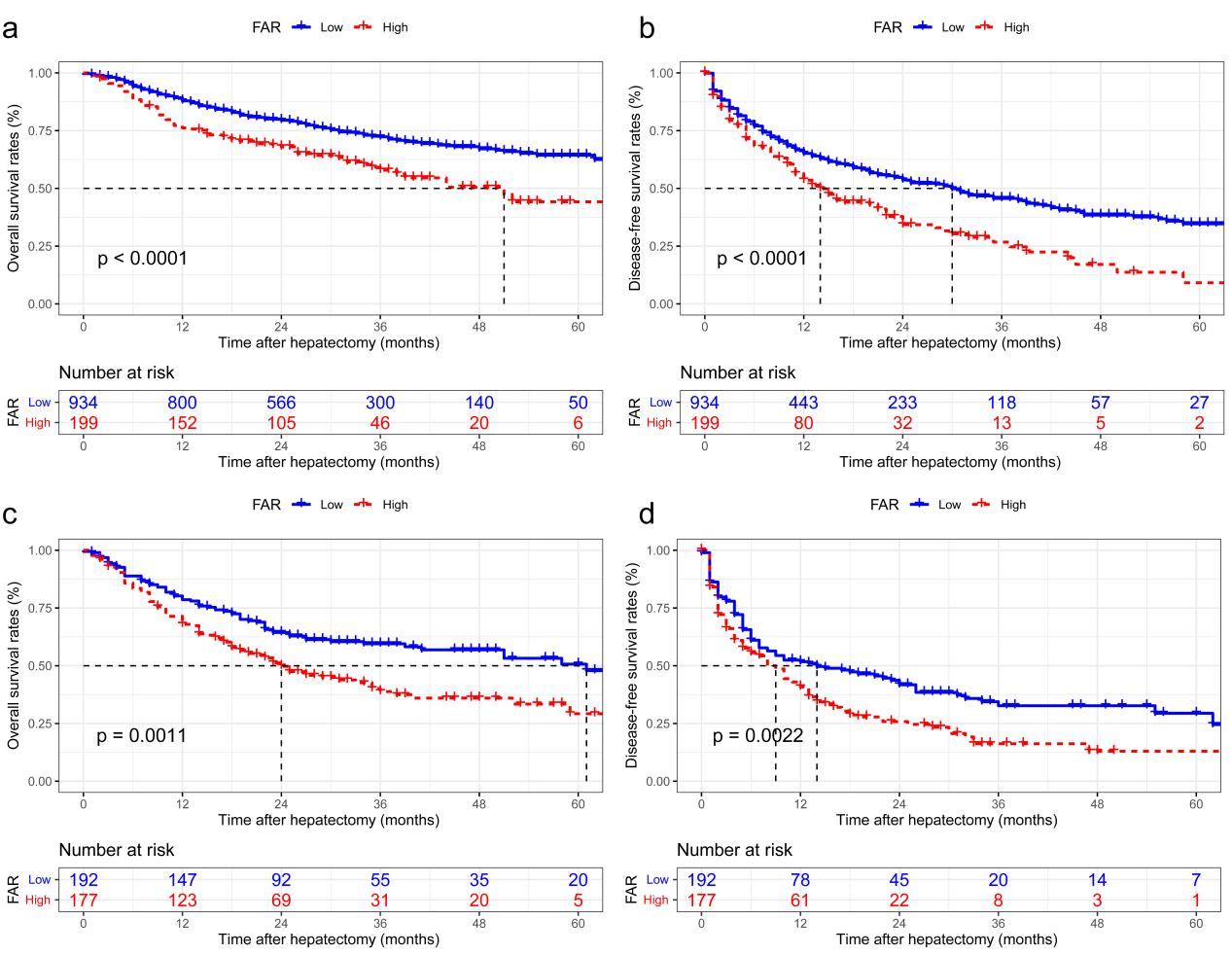
Supplementary Fig 1.** The OS and DFS of patients with high and low FAR were subgroup analyzed according to NLR level. (**a)** OS in patients with low NLR (p *<* 0.001); **(b)** DFS in patients with low NLR (p *<* 0.001); **(c)** OS in patients with high NLR (p *<* 0.001); and **(d)** DFS in patients with high NLR (p *<* 0.001).

Abbreviations: OS overall survival; DFS disease-free survival; FAR, fibrinogen- albumin ratio; NLR, neutrophil–lymphocyte ratio.


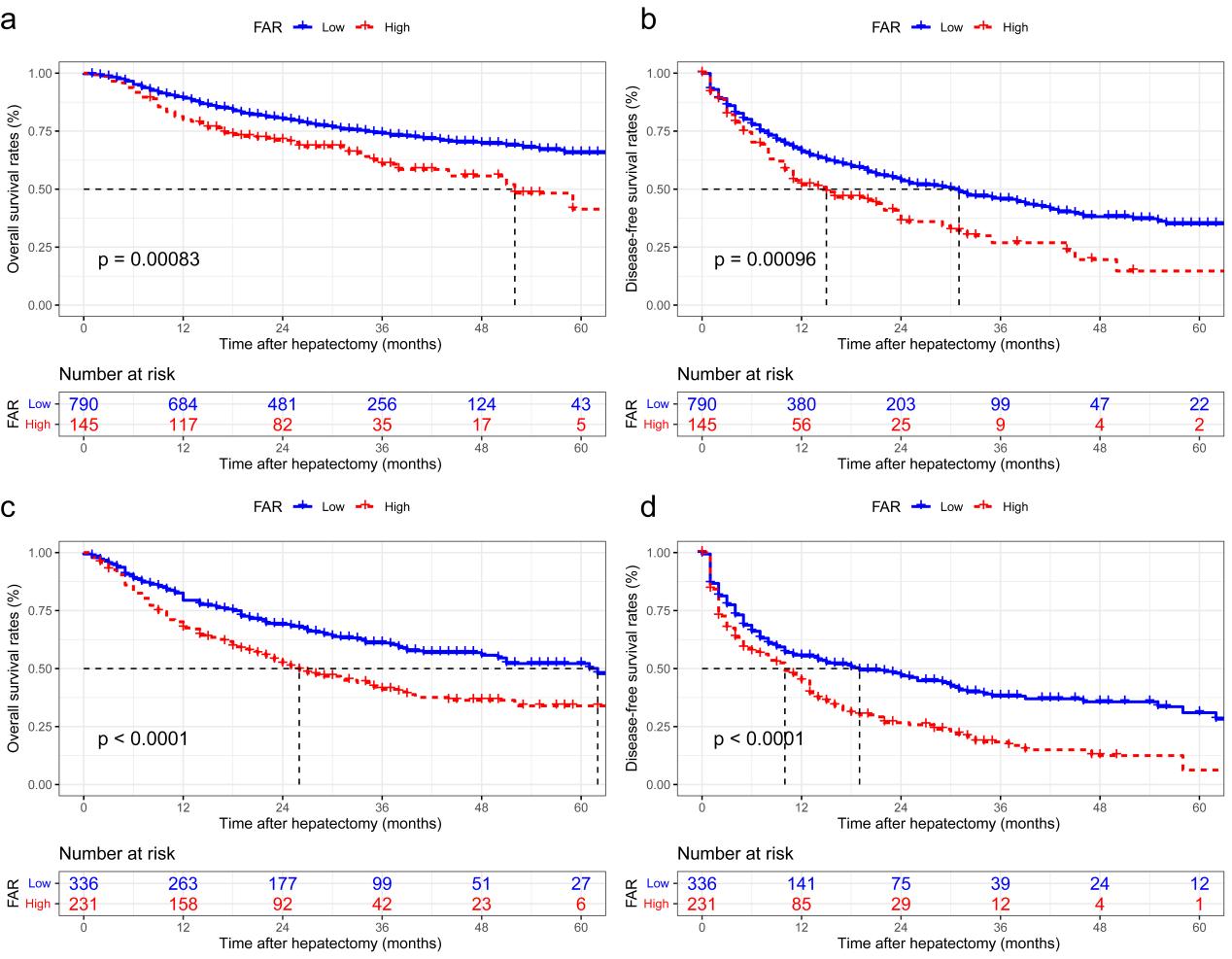


**Supplementary Fig 2.** The OS and DFS of patients with high and low FAR were subgroup analyzed according to MLR level. (**a)** OS in patients with low MLR (p *<* 0.001); **(b)** DFS in patients with low MLR (p *<* 0.001); **(c)** OS in patients with high MLR (p *<* 0.001); and **(d)** DFS in patients with high MLR (p *<* 0.001).

Abbreviations: OS overall survival; DFS disease-free survival; FAR, fibrinogen- albumin ratio; MLR, monocyte-lymphocyte ratio.


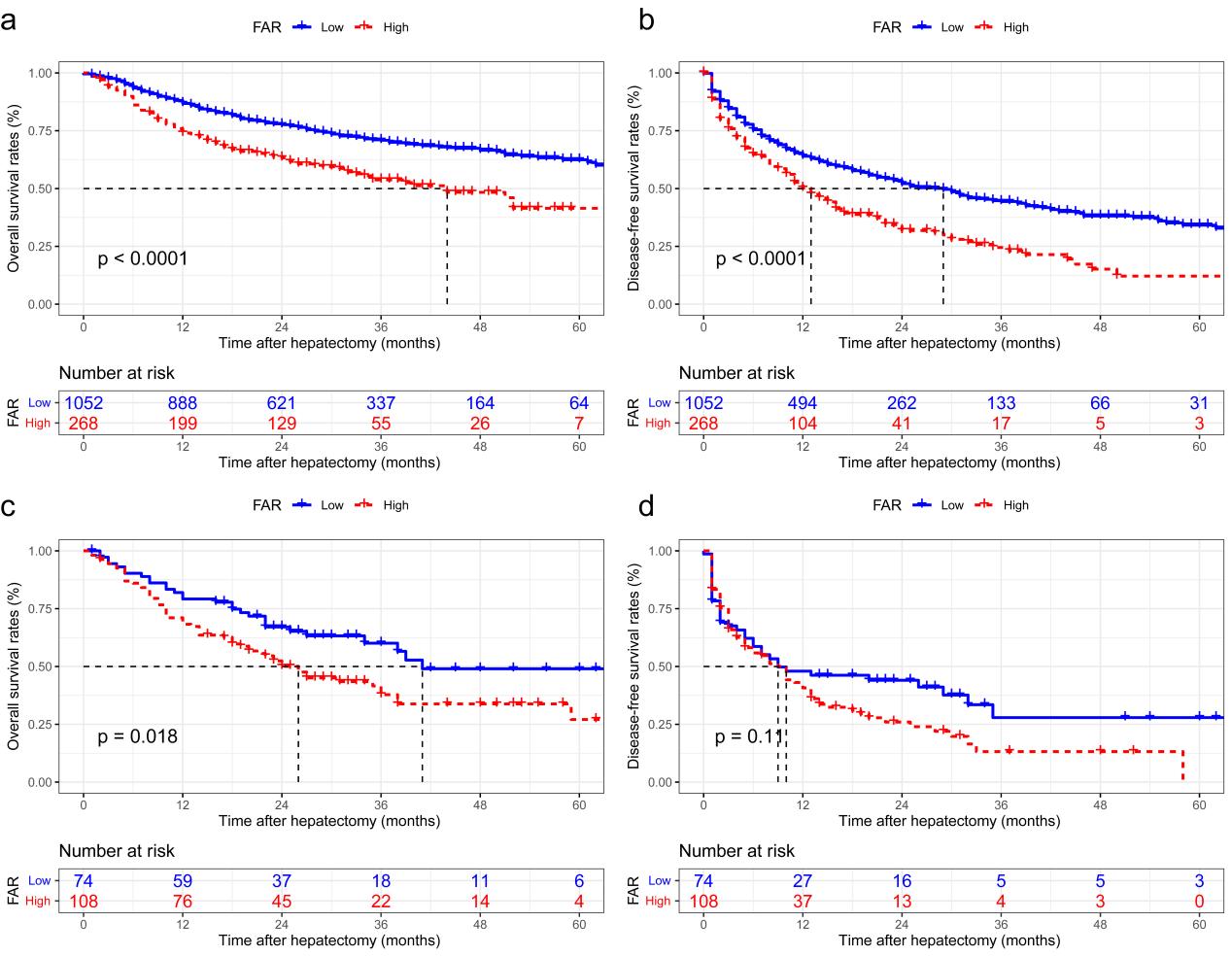


**Supplementary Fig 3.** The OS and DFS of patients with high and low FAR were subgroup analyzed according to MLR level. (**a)** OS in patients with low PLR (p *<* 0.001); **(b)** DFS in patients with low PLR (p *<* 0.001); **(c)** OS in patients with high PLR (p *<* 0.001); and **(d)** DFS in patients with high PLR (p *<* 0.001).

Abbreviations: OS overall survival; DFS disease-free survival; FAR, fibrinogen- albumin ratio; PLR, platelet–lymphocyte ratio.


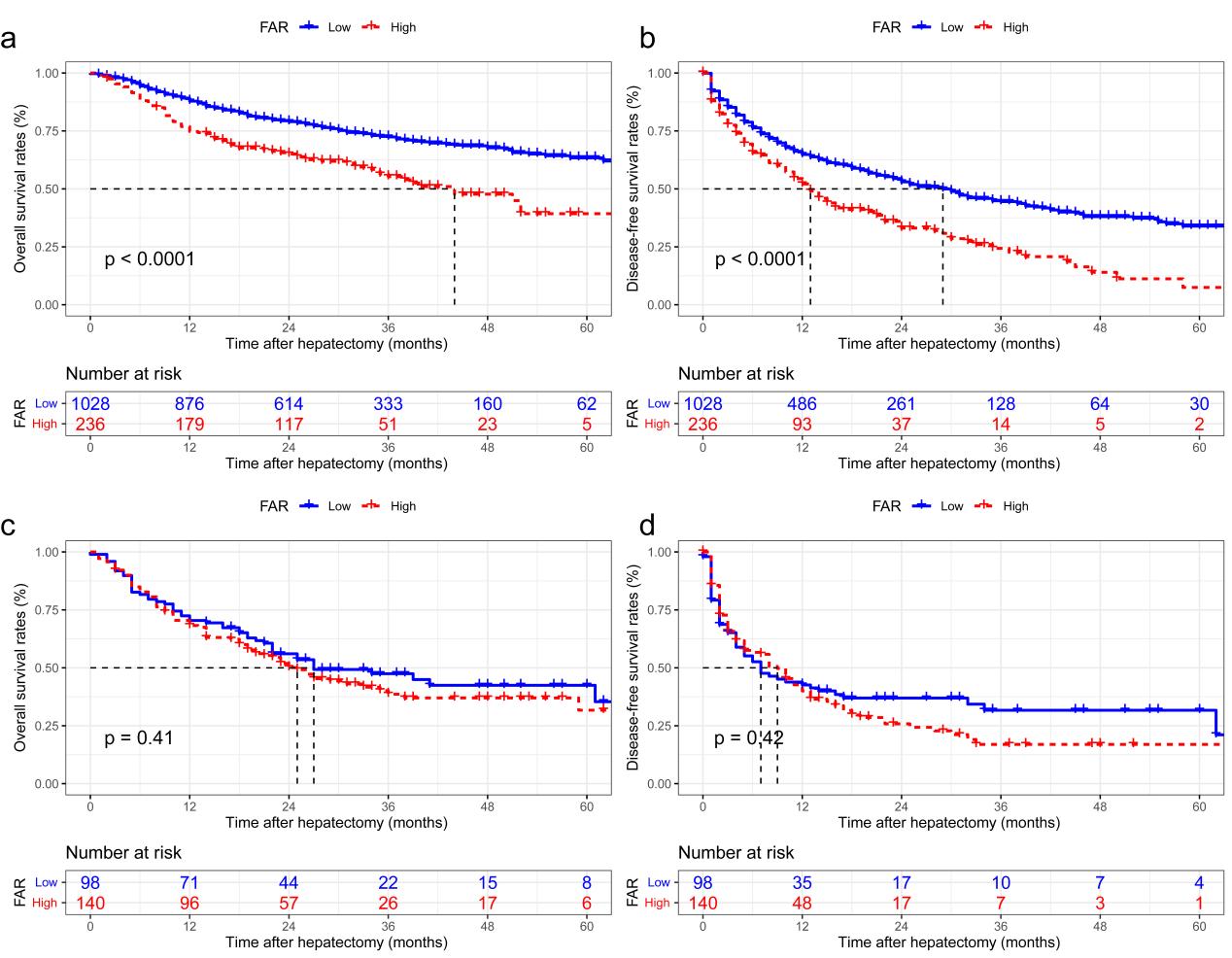


**Supplementary Fig 4.** The OS and DFS of patients with high and low FAR were subgroup analyzed according to SII level. (**a)** OS in patients with low SII (p *<* 0.001); **(b)** DFS in patients with low SII (p *<* 0.001); **(c)** OS in patients with high SII (p *<* 0.001); and **(d)** DFS in patients with high SII (p *<* 0.001).

Abbreviations: OS overall survival; DFS disease-free survival; FAR, fibrinogen- albumin ratio; SII, systemic immune–inflammation index.
